# Lactylation: the metabolic–immune hub in autoimmune diseases

**DOI:** 10.3389/fimmu.2025.1651923

**Published:** 2025-12-18

**Authors:** Qingqing Xia, Dantong Sun, Ke Wan, Chengcheng Liu, Han Shu, Miao Wang, Tongsheng Zhou, Ying Chen, Xue Yang, Xiao-Feng Li, Biao Song, Facai Wang

**Affiliations:** 1 School of Pharmacy, Anhui Medical University, Inflammation and Immune Mediated Disease Laboratory of Anhui Province, The Key Laboratory of Anti-inflammatory and Immune Medicines, Ministry of Education, Hefei, China; 2 Department of Pharmacy, Lu’an Hospital of Anhui Medical University, Lu’an People’s Hospital of Anhui Province, Lu’an, China; 3 School of Pharmacy, The First Affiliated Hospital of Anhui Medical University, Anhui Medical University, Hefei, China; 4 Department of Orthopedics, the Second Affiliated Hospital of Anhui Medical University, Hefei, China

**Keywords:** autoimmune diseases, immune cells, immunometabolism, lactate modification, therapeutic targets

## Abstract

In recent years, lactate modification, as an emerging post-translational modification mechanism, has attracted increasing attention for its role in the regulation of the immune system. Autoimmune diseases are a category of complex disorders characterized by abnormal attacks of the immune system on self-tissues. The limitations of traditional treatments have made the search for new therapeutic targets a hot topic in research. Lactate modification plays a significant role in the development and progression of autoimmune diseases. It can modulate the activation and function of T cells, B cells, macrophages, and dendritic cells, thereby influencing inflammatory and autoimmune responses. In diseases such as experimental autoimmune uveitis (EAU), rheumatoid arthritis (RA), and systemic lupus erythematosus (SLE), lactate modification is closely related to disease progression and can exert its effects by regulating key signaling pathways and cytokine networks. Research on lactate modification as a therapeutic target has also made certain progress, providing new ideas for the treatment of autoimmune diseases. However, there are still many challenges to be faced, such as the development of specific inhibitors, the evaluation of potential side effects, and the feasibility of clinical application. The important regulatory role of lactate modification in autoimmune diseases offers a new target for treatment. Future research needs to further explore its specific mechanisms in the immune system, optimize therapeutic strategies, and assess its clinical application prospects, in order to bring breakthrough progress to the treatment of autoimmune diseases.

## 1 Introduction

**Figure 1:**
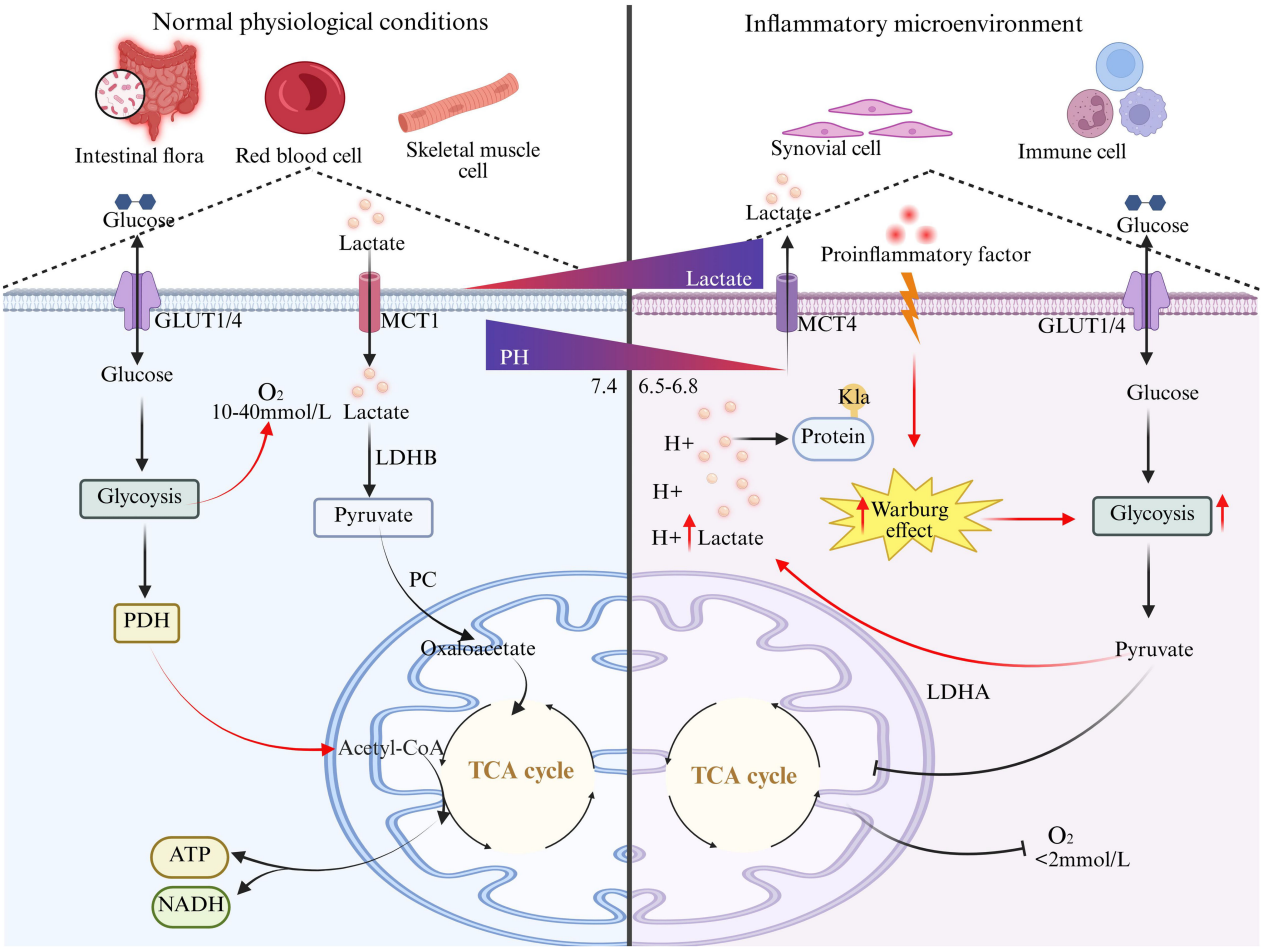
Lactate metabolism in autoimmune inflammation and normal physiology. Under normal physiological conditions, lactic acid originates from the basal glycolysis of skeletal muscle and red blood cells, and is transported by MCT for energy supply or gluconeogenesis, slightly regulating the Th17/Treg balance, with minimal lactylation. In autoimmune inflammation, hypoxia and pro-inflammatory factors drive T cells/macrophages/fibroblasts to burst the Warburg effect, causing a 5-10-fold increase in glycolysis and lactic acid accumulation. Through the pro-inflammatory axis and the immunosuppressive axis, the microenvironment is reshaped, and pathological-level lactylation maintains chronic inflammation, transforming lactic acid from a metabolic waste into a core immune signaling molecule.

Lactate, a core glycolytic metabolite, shows distinct origins and dynamics under physiological versus pathological conditions (**Figure 1**). Under normal conditions, lactate is mainly produced by skeletal muscle during exercise, red blood cells, and gut microbiota via basal glycolysis, maintained at 1–2 mmol/L. It is taken up by organs like the liver and heart through the “lactate shuttle” to sustain metabolic balance (1). Here, lactate acts as an energy intermediate that mildly regulates the Th17/Treg balance, while lactylation plays only minor roles in basic metabolic regulation (2).

In the autoimmune inflammatory microenvironment, hypoxia and cytokines drive infiltrating immune and stromal cells to boost glycolysis via the Warburg effect. This metabolic shift sharply elevates lactate to 10–40 mmol/L and acidifies the local milieu (pH ~6.5-6.8). Such metabolic reprogramming not only supplies rapid energy but also creates a vicious cycle of lactate accumulation and acidification, directly contributing to the initiation and persistence of autoimmune responses.

It is important to note that the abnormal production of lactate is bidirectionally associated with the occurrence and development of autoimmune diseases. On the one hand, the hypoxia and cytokine storm in the inflammatory microenvironment, by activating transcription factors such as HIF-1α and NF-κB, force immune cells to transform into a glycolysis-dependent phenotype (3). On the other hand, excessive lactate reshapes immune cell functions through acidification of the microenvironment, metabolic competition, and lactylation. For example, in rheumatoid arthritis, the high glycolytic activity of synovial fibroblasts leads to lactate accumulation, which stabilizes HIF-1α to promote IL-23 secretion, driving Th17 cell differentiation and the release of IL-17A, thereby exacerbating joint destruction (4).

At the same time, lactate inhibits the mTOR signaling and glucose uptake of CD8^+^T cells, weakening their cytotoxic function (5), and induces M2-type macrophage polarization through the GPR81 receptor, forming an immunosuppressive microenvironment (6). This “pro-inflammatory-immunosuppressive” double-edged sword effect makes lactate a key node connecting metabolic abnormalities and immune imbalances (7).

The discovery of lactylation has unveiled a novel regulatory mechanism of lactate at the epigenetic level. In SLE, histone H3K18 lactylation (H3K18la) recruits methyltransferase 3 (METTL3, a key enzyme catalyzing RNA N^6^-methyladenosine methylation), thereby promoting N^6^-methyladenosine modification of acyl-CoA synthetase long-chain family member 4 (ACSL4) mRNA (8, 9).In a psoriasis model, lactylation of pyruvate kinase M2 (PKM2) inhibits its nuclear translocation, consequently reducing the production of inflammatory cytokines such as IL-1β and IL-6 (6), indicating that lactylation can reprogram the intensity of inflammatory responses through modification of metabolic enzymes. Interventional strategies targeting lactate metabolism, such as lactate dehydrogenase A (LDHA) inhibitors and monocarboxylate transporter 1/4(MCT1/4) blockers, have demonstrated efficacy in experimental models (10, 11). Meanwhile, targeting lactylation-catalyzing enzymes such as p300/CBP or specific modification sites offers new therapeutic directions for autoimmune diseases (12).In summary, lactate metabolism serves a triple role in autoimmune diseases as “fuel, signal, and modulator”. Elucidating its cell-specific mechanisms is expected to provide critical clues for therapeutic breakthroughs.

## 2 History of lactate metabolism and lactylation research

Lactylation is a newly identified post-translational modification that primarily targets lysine residues. It is catalyzed by lactate—a key glycolytic product—which conjugates with lysine via acyl transfer (13). Prevalent in highly glycolytic cells such as cancer cells and metabolically active tissues, lactylation modulates protein function, stability, localization, and interactions (14). As an emerging regulatory mechanism, it is gaining attention in metabolic and disease research (15).

**Figure 2:**
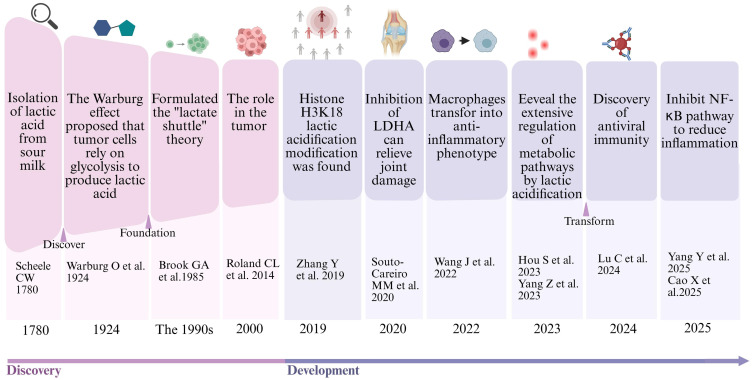
Evolution of protein lactylation research. In 2019, Zhang Rugang’s team discovered histone lactylation (Nature), pioneering a new field of metabolic-epigenetic regulation. From 2020 to 2023, the research expanded to non-histone targets (PKM2/cGAS), revealing their pathological roles in immune metabolic diseases. Currently, the focus is on targeted intervention strategies to promote translational research.

The research landscape has undergone a fundamental shift, evolving from the perception of lactate as a “metabolic waste” to its recognition as a regulatory molecule (**Figure 2**). Early investigations laid the groundwork: from Scheele’s isolation of lactic acid in 1780 (16), to the Warburg effect linking it to tumor metabolism in 1924 (17), followed by findings in the 1990s showing its suppression of T-cell proliferation (18, 19), and its role in establishing an immunosuppressive niche by tumor-associated macrophages in the 2000s (20). The year 2010 provided the first hint of signaling function when lactate was found to activate GPR81 and curb lipolysis (21).

A true paradigm shift occurred in 2019, when Yingming Zhao’s team made the landmark discovery of histone H3K18 lactylation, demonstrating that lactate can directly modify chromatin and promote gene expression, thereby completely overturning its previous perception as “metabolic waste” (22). This ushered in a period of rapid expansion, revealing the central role of lactylation in immunometabolism: In 2020, LDHA-driven lactylation was linked to heightened inflammation in RA CD8^+^T cells (5); 2022 saw PKM2-K62 lactylation block nuclear entry, skewing macrophages toward an anti-inflammatory state (6); 2023 witnessed Ikzf1-K164 lactylation driving Th17 differentiation (23) alongside the mapping of a pan-lactylome with 92% of sites on non-histone proteins (24).

The research frontier has now extended into clinical translation. Lu Chun’s 2024 ALKBH5 lactylation study unveiled its role in antiviral activation (25); Dalian Medical University used machine learning to pinpoint UC-specific lactylation genes, generating a diagnostic AUC of 0.973 and predicting regorafenib targeting; and in 2025, Cao Xuedao demonstrated that the GLO2 substrate SLG non-enzymatically lactylates to suppress NF-κB and relieve inflammation (26, 27). The dual role of lactate in immunity has also been elucidated: in chronic inflammation, it exacerbates autoimmunity by enhancing Th17 differentiation and inhibiting Treg function (23); in acute inflammation, it induces M2 macrophage polarization and inhibits the NLRP3 inflammasome to mitigate damage (28). Future research will focus on developing lactylation enzyme-targeted therapies, spatiotemporally specific editing tools, and precision diagnostic and therapeutic strategies based on lactylation biomarkers, promoting the clinical translation of anti-tumor drugs in autoimmune diseases. This series of milestone events not only reshapes the central position of lactate in immunometabolism but also provides a new perspective for breaking through existing therapeutic bottlenecks.

Autoimmune diseases such as rheumatoid arthritis, systemic lupus erythematosus, and multiple sclerosis (29, 30) are characterized by immune cell dysfunction and chronic inflammation. These diseases are typically manifested by the breakdown of immune tolerance, leading to the formation of autoreactive T cells and B cells, and the production of autoantibodies against self-antigens. These autoantibodies and immune cells cause organ damage, chronic inflammation, and tissue destruction (31), severely affecting the quality of life of patients (32). The pathogenesis of autoimmune diseases is complex, involving genetics (33), environment (34), immune regulation (35) and other aspects. It is estimated that 7.6% to 9.4% of the global population currently suffers from autoimmune diseases, with a higher incidence in women than in men (36). Re-establishing immune tolerance will be the next research hotspot, but there are still many unmet clinical needs in the treatment of various autoimmune diseases. Elevated lactate levels in autoimmune diseases (37) underscore the significance of lactylation—a key mechanism through which lactate modulates immune cell function and disease progression, highlighting its potential as a therapeutic target by intervening in this modification process (38).

## 3 Metabolic regulation, molecular mechanisms, and functions of lactylation

Lactylation, as a newly discovered lysine covalent modification, has achieved significant progress in understanding its molecular mechanisms and biological functions. This modification is mediated by lactyl-CoA and originates from glycolytic “Warburg effect”-derived lactate, gut microbiota metabolites, and hypoxia-induced metabolic reprogramming (39, 40). Through dynamic regulation of histone and non-histone functions (41), lactylation influences chromatin remodeling, transcriptional activity, and protein stability, thereby participating in gene expression regulation and the establishment of immunometabolic networks (37, 42, 43). Studies have revealed that lactylation integrates microenvironmental cues through a “metabolism–epigenetics–immunity” axis, serving as a pivotal regulator of inflammatory polarization, immune-cell control such as macrophage phenotype switching) and the dynamic progression of disease (44).

At the molecular level, lactylation can be catalyzed by “writer” enzymes such as p300, GCN5, and HBO1 using lactyl-CoA as a donor (45),with p300 being the first identified histone lactyltransferase (22). Alternatively, under pathological conditions such as hypoxia, lactylation can also occur through a non-enzymatic pathway mediated by lactyl-glutathione (46). Based on the modification sites, lactylation is classified into histone lactylation, including H3K18la and H3K14la, and non-histone lactylation. Histone lactylation regulates gene transcription by altering chromatin structure (47, 48), whereas non-histone lactylation modulates the functions of various proteins, such as metabolic enzymes and transcription factors. For instance, AARS1-mediated lactylation of p53 regulates cell proliferation (49, 50).

**Figure 3:**
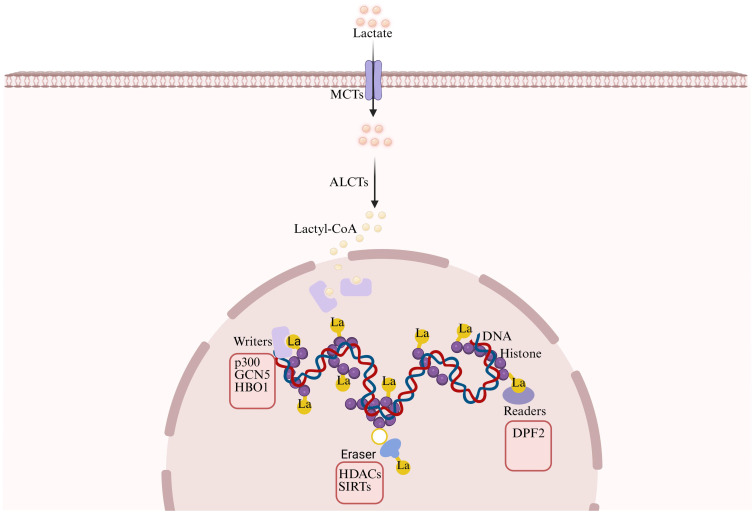
Molecular machinery of lactylation. The lactylation process is precisely regulated by three types of regulatory proteins. Writer enzymes (e.g., p300, GCN5, HBO1) catalyze the modification using lactyl-CoA as a donor substrate; under pathological conditions such as hypoxia, non-enzymatic modification can also occur via lactyl-glutathione. Eraser enzymes (e.g., SIRT1) are responsible for removing the modification. Reader proteins(e.g., DPF2) recognize specific lactylation marks (e.g., H3K14la) and recruit downstream effector complexes to regulate gene expression.

This modification process is finely regulated by three types of regulatory proteins (**Figure 3**): “writers” such as p300, GCN5, and HBO1 catalyze the modification (51), with HBO1 participating in tumor progression through mediating H3K9la; “erasers” such as SIRT1 remove the modification, and its activator resveratrol can improve cardiac function (52); “readers” such as DPF2 regulate gene expression by recognizing modification sites like H3K14la (53). Notably, lactylation exhibits complex interactions with other post-translational modifications such as acetylation and phosphorylation, either competing for the same sites or synergistically regulating cellular processes (54, 55).

In hypoxic microenvironments, lactylation participates in metabolic reprogramming by stabilizing HIF-1α (56). In tumor drug resistance, lactylation at K76 of IGF2BP3 protein drives serine metabolic reprogramming and promotes liver cancer drug resistance by enhancing its binding capacity to N6-methyladenosine-modified mRNA (57). These findings not only expand our understanding of post-translational modification regulatory networks but also provide novel molecular targets for related disease therapies.

## 4 Crosstalk between lactylation and the immune system

Lactylation converts extracellular lactate into an immune command: under health it sustains homeostasis, while in autoimmunity or tumors it triggers dysregulated responses via pathway rewiring, epigenetic shift, and metabolic overhaul.

### 4.1 Impact on the NF-κB pathway

Lactylation directly modulates the expression of pro-inflammatory genes by targeting key components of the NF-κB signaling axis. In macrophages, for example, lactylation of proteins such as Nuclear factor of kappa light polypeptide gene enhancer in B-cells inhibitor, zeta (IκBζ) enhances their stability or transcriptional co-activator capacity, thereby boosting the production of cytokines like IL-6 and IL-12 (22).This regulatory mechanism amplifies innate immune responses during both infectious and sterile inflammation. Moreover, in autoimmune arthritis models, lactate accumulation within synoviocytes induces protein lactylation that indirectly activates the NF-κB pathway by stabilizing HIF-1α. This drives IL-23/IL-17 axis–mediated inflammation and exacerbates tissue destruction (58).

### 4.2 Regulation of the cGAS–STING pathway

Recent studies have shown that lactylation serves as a crucial metabolic link in innate immunity. In the cytosol, lactate can directly modify key residues on cyclic GMP-AMP synthase (cGAS), impairing its DNA-binding capacity and enzymatic activity, thereby attenuating the type I interferon response mediated by the cGAS-STING pathway (59). This mechanism allows tumor cells to evade immune surveillance within the tumor microenvironment, yet it can also lead to suboptimal antiviral immune responses during viral infections. Moreover, lactylation of the STING protein itself may influence its oligomerization and downstream signal transduction, thereby finely tuning both the intensity and duration of pathway activation.

### 4.3 Epigenetic regulation of immune cell differentiation and function

Histone lactylation is a key epigenetic mechanism that shapes the gene-expression landscape in immune cells. In macrophages, the promoter regions of M2-polarization marker genes such as Arg1 show elevated H3K18la modification, which directly boosts their transcription and thereby drives macrophage differentiation toward an anti-inflammatory, pro-reparative phenotype (60). Conversely, in Th17 cells, lactylation enhances chromatin accessibility at the RORγt and IL-17 loci, thereby promoting their differentiation and pathogenicity. Simultaneously, this modification suppresses the function of regulatory T cells, disrupts immune tolerance, and precipitates uncontrolled inflammatory responses (61).

### 4.4 Regulation of autoantibody production

In autoimmune disorders such as SLE, lactylation promotes autoantibody generation through multiple mechanisms. On one hand, it modifies histones or transcription factors, thereby enhancing the expression of genes associated with plasma-cell differentiation and antibody secretion. On the other hand, recent work shows that lactylation can inhibit the enzymatic activity of metabolic enzymes, for example by lactylating GAPDH, which increases citrullination of self-antigens. This process supplies additional autoantigenic material, breaches B-cell tolerance, and ultimately triggers the production of high-titer autoantibodies (59).

### 4.5 Metabolic reprogramming and immune function crosstalk

Lactylation creates a self-amplifying, metabolic-functional positive-feedback loop in immune cells. Highly glycolytic immune subsets such as activated T cells and M1 macrophages produce abundant lactate. In turn, lactate-driven lactylation stabilizes HIF-1α and enhances the activity of glycolytic enzymes such as PKM2 and LDHA, thereby reinforcing the Warburg effect while concurrently suppressing mitochondrial oxidative phosphorylation (1). This metabolic reprogramming not only furnishes rapidly proliferating, highly functional immune cells with biosynthetic precursors; the resulting lactate and the ensuing acidification of the microenvironment further suppress the cytotoxic activity of CD8^+^T cells and NK cells via lactylation, thereby fostering an immunosuppressive milieu.

In summary, Lactylation links metabolism to immunity, calibrates signaling and epigenetics, and mirrors disease activity in RA and SLE, offering both biomarker and therapeutic leverage.

## 5 Lactylation and immune cell dysfunction

**Figure 4:**
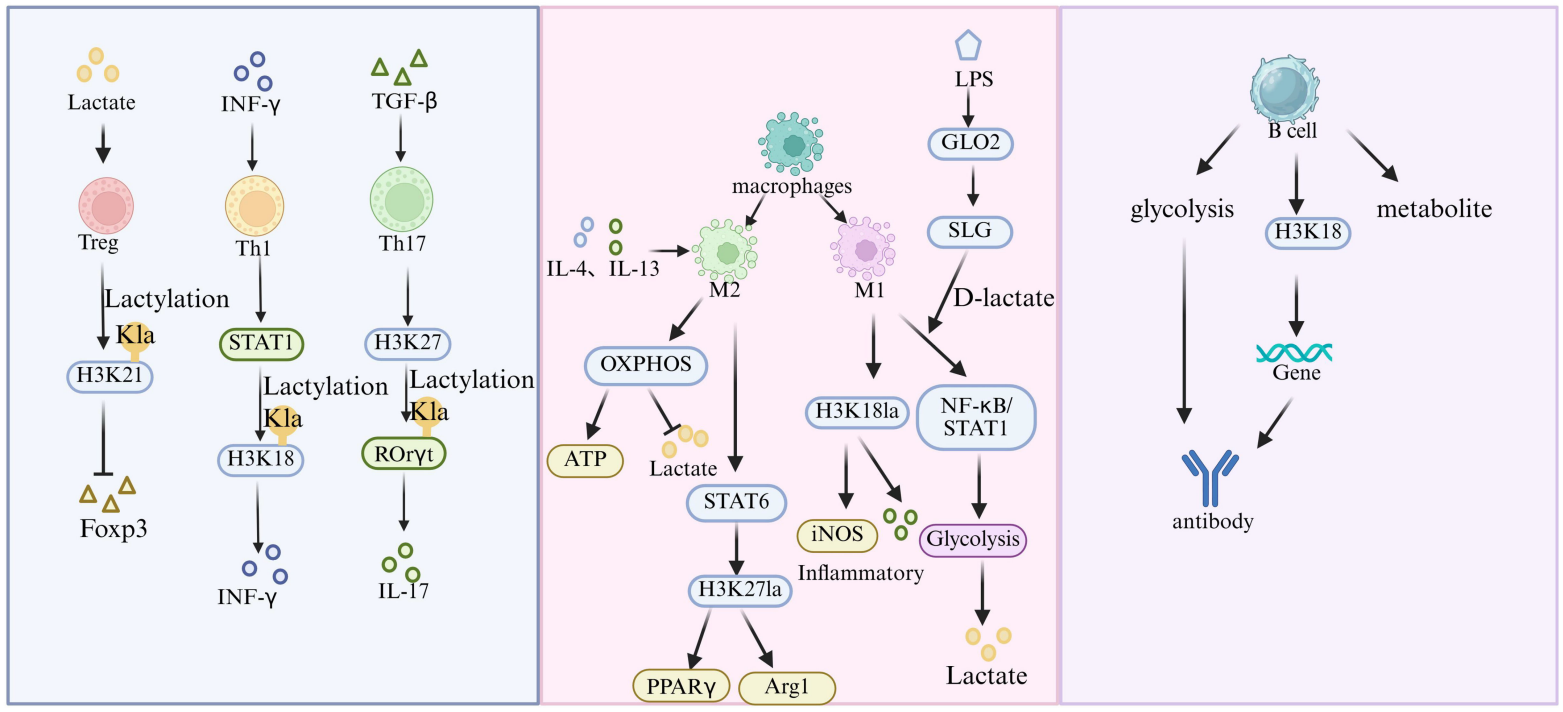
Lactylation and immune cell dysfunction. The diagram illustrates the effects of lactate on different immune cells. In T cells, it regulates the Th17/Treg balance by altering metabolism and modifying key transcription factors, thereby affecting inflammation and immune tolerance. For macrophages, lactylation influences polarization, particularly M1 pro - inflammatory phenotype, via metabolic molecule SLG accumulation and the GLO2/SLG/D - lactate axis, impacting inflammation. In B cells, lactylation modulates metabolism and antibody secretion, affecting autoantibody production through metabolic regulation and epigenetic mechanisms.

Lactylation, as an emerging post-translational modification of proteins, has profound effects on the functions and activities of immune cells (**Figure 4**).

### 5.1 T cells

T cells play a critical role in the immune system, with CD4^+^T-cell subsets including T helper 1, Th17, and regulatory T cells playing pivotal roles in the pathogenesis and progression of autoimmune diseases. Th1 cells contribute to cellular immune responses and drive inflammatory reactions through the secretion of IFN-γ; Th17 cells secrete IL-17, promoting inflammation and autoimmune damage; whereas Treg cells maintain immune tolerance by producing inhibitory cytokines such as IL-10 and TGF-β (58). Lactylation can regulate the balance between Th1/Th17 and Treg cells through multiple mechanisms, influencing the expression of key cytokines and the function of transcription factors (62).Within the inflammatory microenvironment, lactate accumulation induces metabolic reprogramming in T cells, enhancing glycolysis and further promoting lactate production. This metabolic shift facilitates the differentiation of Th1 and Th17 cells while simultaneously suppressing Treg function (47), Furthermore, lactylation modulates T-cell differentiation by regulating histone modification states—for instance, by elevating histone H3K18 lactylation levels (42) and suppressing Foxp3 transcription. This modification can also directly target transcription factors; it enhances Th17 differentiation by modulating RORγt activity (63) and may impair Treg function by influencing Foxp3 protein stability (64).

### 5.2 Macrophages

Lactylation plays a key role in macrophage polarization by participating in the regulation of pro-inflammatory M1-type macrophage function and enhancing the release of inflammatory factors such as TNF-α and IL-6 (13). Studies have shown that immune activation leads to a sharp decrease in the activity of the methylglyoxal metabolic enzyme GLO2 within macrophages, resulting in the accumulation of its substrate S-D-lactoylglutathione (SLG) (65). Through a non-enzymatic reaction, SLG specifically induces D-lactylation at the K310 site of the NF-κB subunit RelA, thereby inhibiting NF-κB activation and reducing the secretion of pro-inflammatory cytokines (66). This modification negatively regulates inflammatory signaling pathways, forming a GLO2/SLG/D-lactylation metabolic feedback axis (27). This axis helps maintain immune homeostasis by modulating metabolite accumulation, and its dysregulation may drive excessive M1 polarization of macrophages, exacerbating inflammatory responses.

In anti-inflammatory M2 macrophages, IL-4/IL-13 signaling induces oxidative phosphorylation (OXPHOS) via STAT6, thereby reducing intracellular lactate levels. Meanwhile, histone H3K27 lactylation (H3K27la) activates anti-inflammatory genes such as PPARγ and Arg1 to maintain immune homeostasis (22). However, in specific microenvironments, IL-4 can drive macrophages toward a pro-inflammatory phenotype (M2INF) through the glycolysis/HIF-1α axis, establishing a “bistable switch” for immune balance (67).

### 5.3 B cells

Lactylation affects the production of autoantibodies by influencing B cell metabolism. B cells play a key role in autoimmune diseases, and their abnormal activation and excessive secretion of autoantibodies are important pathological features of autoimmune diseases. lactylation affects their function by influencing the metabolic state and signaling transduction of B cells. The regulatory effect of lactylation on B cell metabolism is closely related to the production of autoantibodies. Specifically, this modification can regulate the metabolic pathways of B cells, thereby affecting their function and antibody secretion levels. Studies have confirmed that lactylation can affect the glycolysis and oxidative phosphorylation pathways of B cells (68), thereby regulating the production of autoantibodies. In addition, lactylation can also regulate B cell function through epigenetic mechanisms. For example, lactylation can enhance the modification level of histone H3K18 (69), thereby promoting the expression of genes related to B cell differentiation and antibody secretion. In terms of immune metabolic regulation, lactylation can regulate the accumulation of metabolic molecules within B cells, forming an immune metabolic regulatory feedback loop. When this regulatory loop is disrupted, it may lead to the overactivation of B cells and the excessive secretion of autoantibodies, thereby exacerbating the pathological process of autoimmune diseases.

## 6 Regulation of immune responses by lactylation

It has been found that the downregulation of the methylglyoxal metabolic enzyme GLO2 in immune cells leads to the accumulation of the metabolic molecule SLG (27), which in turn induces D-lactoylation modification of cytoplasmic proteins and negatively regulates inflammatory responses. This modification regulates immune signaling pathways such as NF-κB to inhibit the secretion of pro-inflammatory factors and maintain immune homeostasis. In the tumor microenvironment, lactylation regulates the modification level of histone H3K18 to inhibit the anti-tumor activity of tumor-associated macrophages (TAMs) (70). In addition, lactylation also regulates the accumulation of metabolic molecules to enhance immune suppression signals and promote the generation of Tregs. lactylation can also regulate the activity of cGAS protein to inhibit the innate immune response it mediates. It has been found that AARS2-mediated cGAS lactylation inhibits the synthesis of cGAMP (71), thereby weakening the innate immune response.

In SLE, lactylation of cGAS induced by mitochondrial DNA activates the interferon response, promoting the production of autoantibodies (59). In addition, lactylation regulates the metabolic pathways of B cells to affect the secretion of autoantibodies. lactylation can regulate the metabolic state and signaling transduction of immune cells, affecting autoimmune responses. For example, lactylation regulates the expression of histone modifications (72), enhancing the expression of genes related to B cell differentiation and antibody secretion.

Lactylation regulates the accumulation of metabolic molecules (73), to construct a complex immune metabolic regulatory feedback loop. When this regulatory loop is disrupted, it may lead to the overactivation of immune cells and the disruption of immune tolerance balance in the body. lactylation can also negatively regulate inflammatory signaling pathways such as NF-κB (74), to inhibit the secretion of pro-inflammatory factors and maintain immune homeostasis. lactylation can also regulate the accumulation of metabolic molecules within immune cells to affect the activation state of immune cells (8). These research results indicate that lactylation has important regulatory functions in the immune response process, with dual regulatory effects on immune metabolism and inflammatory signaling pathways, suggesting that metabolic enzymes such as GLO2 may become potential targets for regulating immune responses (75), providing a theoretical basis for the development of novel therapies for inflammatory diseases.

## 7 In-depth studies of lactylation in specific autoimmune diseases

### 7.1 Experimental autoimmune uveitis

EAU is a classic animal model widely used in the study of the pathogenesis of human uveitis. Uveitis is a complex ocular inflammatory disease involving the activation of various immune cells and the amplification of inflammatory responses.

In the pathogenesis of EAU, lactylation plays a crucial role by regulating the differentiation and function of immune cells. In the EAU mouse model, CD4^+^T cells are the main pathogenic cell type (76), which can differentiate into various effector cell subsets, including Th1, Th2, and Th17 cells. Th17 cells play a central role in the pathogenesis of EAU.

Lactylation significantly affects the differentiation of CD4^+^T cells by regulating the activity of transcription factors. With the progression of EAU, the lactate content in mouse spleen tissues and CD4^+^T cells is significantly upregulated (77), promoting the increase in intracellular lactylation levels. This modification significantly promotes the pro - inflammatory polarization of Th17 cells by enhancing the binding ability to the IL-17A/RORγt promoter, driving autoimmune inflammation (78). In addition, lactylation of PKM2 at K62 in M1 macrophages can inhibit its nuclear translocation, maintain glycolysis, and promote the secretion of IL-1β/TNF-α, exacerbating tissue damage (6). Further mechanistic studies have shown that the Lys164 site of the transcription factor Ikzf1 undergoes high levels of lactylation. This modification directly regulates the expression of Th17-related genes such as Runx1, Tlr4, IL-2, and IL-4, significantly promoting the differentiation of Th17 cells (79). lactylation also affects the activation and function of other immune cells, including macrophages, dendritic cells, and natural killer cells, thereby further exacerbating the inflammatory response in EAU.

In addition to its regulatory effects on immune cells, lactylation may also affect the pathogenesis of EAU by altering the metabolic state of ocular tissues. The metabolic state of ocular tissues in patients with uveitis is closely related to the intensity and duration of the inflammatory response (80). The increase in lactate, the end product of the glycolysis pathway, changes the metabolic microenvironment of ocular tissues, thereby promoting the continuous progression of the inflammatory response.

In the EAU mouse model, with the progression of the disease, the lactate content and lactylation levels in spleen tissues and CD4^+^T cells show an increasing trend (81), which is positively correlated with the pathological manifestations of EAU. Intervening in the level of lactylation, for example, using glycolysis inhibitors or lactylation antibodies, can significantly affect the differentiation of Th17 cells and alleviate or worsen the pathological manifestations of EAU (82). Studies have found that there is a close relationship between lactylation levels and disease activity in EAU. In EAU mice, individuals with higher lactylation levels often exhibit stronger inflammatory responses and more severe ocular damage (80). he prognosis of EAU mice is also closely related to lactylation levels. Individuals with higher lactylation levels recover more slowly after treatment and are more prone to recurrence. These results suggest that lactylation is not only an important link in the pathogenesis of EAU but may also serve as a potential biomarker for disease monitoring and the development of therapeutic targets. Lactylation of T-bet at an undefined site in Th1 cells can inhibit its activity, reduce IFN-γ secretion, weaken the cellular immune response, and is associated with the pathogenesis of rheumatoid arthritis (83).

### 7.2 Rheumatoid arthritis

RA is a chronic autoimmune disease, mainly characterized by synovial inflammation and proliferation of joints, eventually leading to joint destruction and functional impairment (84).

The potential role of lactylation in RA is mainly reflected in its effects on plasma cells. Plasma cells are the terminal differentiated products of B cells, primarily responsible for antibody production in response to pathogen infections. In autoimmune diseases, the abnormal activation of plasma cells and the excessive production of antibodies are key factors driving the occurrence and progression of diseases. Studies have found that lactylation is highly expressed in plasma cells and is involved in the pathogenesis of RA. Through single-cell RNA sequencing and machine learning techniques, researchers have identified core lactylation-promoting genes that are upregulated in plasma cells of RA patients and are closely related to disease activity (85). These core lactylation-promoting genes promote antibody production and immune complex deposition by regulating the activation and function of plasma cells, thereby exacerbating the inflammatory response and tissue damage in RA. lactylation can also affect the activation and function of other immune cells (86), such as T cells, macrophages, and dendritic cells, which are also involved in the pathogenesis of RA. The abnormal activation of these immune cells leads to persistent synovial inflammation and proliferation, further promoting the occurrence of joint destruction and functional impairment. In fibroblast-like synoviocytes, lactylation of HIF-1α at K32 stabilizes HIF-1α, promotes the secretion of VEGF/MMP3 and the formation of pannus, and exacerbates joint destruction (87). Lactylation of PKM2 at K62 in M1 macrophages inhibits its nuclear translocation, continuously activates glycolysis, and promotes the release of IL-1β/TNF-α, driving inflammatory progression (6). In addition, lactic acidification of the B cell microenvironment can enhance BCR signaling(modification site undetermined), promote autoantibody production, and induce humoral immune abnormalities (88).

There is a close correlation between lactylation and RA disease activity. In RA patients, individuals with higher lactylation levels often exhibit stronger inflammatory responses and more severe joint damage, and lactylation levels are also positively correlated with RA disease activity scores such as DAS28 scores (89). lactylation may serve as a potential biomarker for assessing RA disease activity. To further verify the correlation between lactylation and RA disease activity, researchers have constructed a new diagnostic model - the RA lactylation score (85). This model is based on the expression levels of lactylation-related proteins in the plasma of RA patients and has high predictive performance, which can be used to assess the likelihood of an individual developing RA and predict disease progression and prognosis.

### 7.3 Systemic lupus erythematosus

SLE is a systemic autoimmune disease involving the abnormal activation of immune cells and the production of autoantibodies (90). In Treg cells, lactylation of FOXP3 at K273 hinders its binding to DNA, weakens immunosuppressive functions, and leads to disruption of immune tolerance (27). Lactylation of the B cell BCR complex (mediated by microenvironmental lactic acidification, site undetermined) can enhance signal transduction and promote autoantibody secretion (88). lactylation promotes the progression of SLE by regulating the abnormal activation of type I interferon pathway.

The abnormal activation of the type I interferon pathway is one of the key mechanisms in the progression of SLE. lactylation promotes the overproduction of type I interferon by affecting the activity of interferon regulatory factors such as IRF3, IRF7 and signal transduction molecules such as STAT1, STAT2 (91). This abnormal activation not only leads to the overactivation of immune cells but also exacerbates tissue damage and inflammatory responses.

Lactylation can affect the activation of the type I interferon pathway through multiple pathways (6):

Affecting the activity of transcription factors: lactylation may affect the activity of IRF3 and IRF7 to promote the expression of interferon-related genes.

Regulating the stability of signal transduction molecules: lactylation may affect the stability of STAT1, promoting its accumulation in the nucleus and thereby enhancing the signal transduction of type I interferon.

Regulating the cGAS-STING pathway: cGAS is a cytoplasmic DNA sensor, and its lactylation can inhibit the activity of cGAS, thereby affecting the activation of the cGAS-STING signaling pathway. In SLE patients, lactylation of cGAS may lead to a decrease in its recognition ability for self-DNA, thereby inhibiting the production of type I interferon.

Lactylation can also promote the production of autoantibodies by affecting the metabolism and function of B cells. It has been found that monocarboxylate transporter 1 (MCT1) is upregulated in SLE patients, and it affects B cell antibody class switching and autoantibody secretion by regulating pyruvate metabolism and H3K27 acetylation (72).

In SLE, lactylation is not only involved in the regulation of the type I interferon pathway but is also closely related to multiple characteristics of the disease. High levels of autoantibodies such as anti-dsDNA antibodies in SLE patients are one of the important markers of the disease (92), and lactylation promotes the production of these autoantibodies by affecting the metabolism and function of B cells. Studies have shown that the high expression of MCT1 is closely related to the production of anti-dsDNA antibodies (93) and the aggregation of IgG antibodies in glomeruli. In addition, lactylation exacerbates the inflammatory response in SLE patients by regulating the type I interferon pathway and inflammatory signaling pathways such as the NF-κB pathway (94), lactylation can enhance the nuclear translocation of IRF3 and NF-κB, thereby promoting the secretion of pro-inflammatory cytokines such as TNF-α, IL-6. lactylation also exacerbates tissue damage in SLE patients by affecting cell metabolism and immune cell activation. For example, lactylation can lead to the leakage of mitochondrial DNA, activating inflammatory pathways and thereby causing tissue damage. In addition, lactylation may also affect the function of other immune cells such as macrophages, dendritic cells (95), further exacerbating the progression of SLE. For example, lactylation can inhibit the phagocytic function of macrophages, leading to a decrease in the ability to clear pathogens and thereby increasing the risk of infection.

### 7.4 Research clues of lactylation in other autoimmune diseases

In addition to the aforementioned EAU, RA, and SLE, research clues on lactylation in other autoimmune diseases are also gradually increasing. lactylation has been found to be involved in the activation of immune cells and inflammatory responses in autoimmune thyroid diseases, multiple sclerosis, psoriasis, and other diseases.

In autoimmune thyroid diseases, including Graves’ disease and Hashimoto’s thyroiditis, lactylation is involved in thyroid cell apoptosis and inflammatory responses (96). By intervening in the level of lactylation, the survival and immune function of thyroid cells can be affected, thereby regulating the progression and prognosis of the disease.

Multiple sclerosis is a central nervous system demyelinating disease involving the abnormal activation of immune cells and the disruption of the blood-brain barrier. In patients with multiple sclerosis, lactate levels in cerebrospinal fluid and brain tissues are significantly elevated. This increase in lactate levels is closely related to the activation of immune cells and inflammatory responses. lactylation is involved in the pathogenesis of multiple sclerosis by regulating the differentiation and function of immune cells and affecting the metabolic state of neurons, exacerbating disease progression and damage (97). In patients with multiple sclerosis, lactate levels in cerebrospinal fluid and brain tissue are elevated. Lactylation exacerbates central nervous system inflammation by regulating the differentiation of Th17 cells such as lactylation of Ikzf1 at K164 and neuronal metabolism (78).

Psoriasis is a common chronic skin disease characterized by symptoms such as skin erythema, scaling, and itching. Lactate levels in the skin lesions of patients with psoriasis are significantly elevated (98). This increase in lactate levels is closely related to the abnormal proliferation of keratinocytes and inflammatory responses. Studies have found that lactylation exacerbates the formation and development of skin lesions by regulating the proliferation and differentiation of keratinocytes and affecting the activation and function of immune cells (99) (**Table 1**).

**Table 1:** The regulation of lactylation in cell type and disease.

Cell type	Lactylation target	Regulatory mechanism	Functional impact	Related diseases	References
Th1 cells	T-bet (site to be determined)	Lactylation inhibits T-bet activity → IFN-γ secretion ↓	Weakened cellular immune response	Rheumatoid Arthritis (RA)	(77)
Th17 cells	Ikzf1 K164	Lactylation enhances IL-17A/RORγt promoter binding → Pro-inflammatory polarization ↑	Drives autoimmune inflammation	Multiple Sclerosis, Experimental Autoimmune Uveitis (EAU)	(72)
Treg cells	FOXP3 K273	Lactylation hinders FOXP3-DNA binding → Immunosuppressive function ↓	Breaks immune tolerance	Systemic Lupus Erythematosus (SLE), Type 1 Diabetes	(22)
M1 macrophages	PKM2 K62	Lactylation inhibits PKM2 nuclear translocation → Glycolysis sustained → IL-1β/TNF-α secretion ↑	Promotes tissue damage	RA, Inflammatory Bowel Disease (IBD)	(6)
M2 macrophages	STAT6 K108	Lactylation enhances STAT6 phosphorylation → Arg1/Ym1 expression ↑	Promotes tissue repair	Fibrosis, Wound healing	(72)
B cells	BCR complex (site not determined)	Microenvironmental lactic acidification → Enhanced BCR signaling → Increased autoantibody production	Induces humoral autoimmunity	SLE, Sjogren’s Syndrome	(82)
Fibroblast-like synoviocytes	HIF-1α K32	Lactylation stabilizes HIF-1α → VEGF/MMP3 secretion ↑ → Pannus formation	Exacerbates joint destruction	RA	(81)

### 7.5 Lactylation in immune-mediated diseases

The mechanisms of lactylation in tumors are complex and diverse, especially in tumor immune evasion, metabolic reprogramming, and treatment resistance. For example, in hepatocellular carcinoma (HCC), ABCF1-K430 lactylation activates the HIF1 signaling pathway, promoting tumor growth and metastasis, forming a positive feedback loop and leading to poor prognosis in patients. Tubuloside A (TubA) is a small-molecule drug that targets this site and inhibits the progression of HCC (100). Similarly, AK2-K28 lactylation in HCC damages kinase activity, leading to energy metabolism disruption, enhancing tumor proliferation and invasion, and is associated with poor prognosis and increased risk of thrombosis (24).

In metabolic regulation, high lactate levels in the tumor microenvironment drive lactylation through the Warburg effect. In non-small cell lung cancer (NSCLC), high LDHA expression leads to lactate accumulation, inducing APOC2-K70 lactylation, expanding Tregs, inhibiting CD8^+^T cells, and creating an immunosuppressive microenvironment (50). Lactylation also affects DNA repair; for example, lactylation of MRE11 enhances homologous recombination repair (HRR), causing chemoresistance in gastric cancer (101).

It is worth noting that lactylation regulates key tumor suppressors in cancer. A research group from Soochow University found that AARS1-mediated lactylation of p53 at K120 and K139 sites damages its DNA binding and liquid-liquid phase separation, inhibiting its tumor suppressor function. This mechanism is associated with poor prognosis in cancers with wild-type p53, such as breast cancer and liver cancer (50). In animal models, targeting this pathway with β-alanine to compete for AARS1 binding reduces p53 lactylation and shows potential for enhancing chemotherapy. These findings highlight the central role of lactylation in cancer and provide new molecular insights for cross-disease mechanism research (such as the interaction between autoimmune diseases and the tumor immune microenvironment).

**Figure 5:**
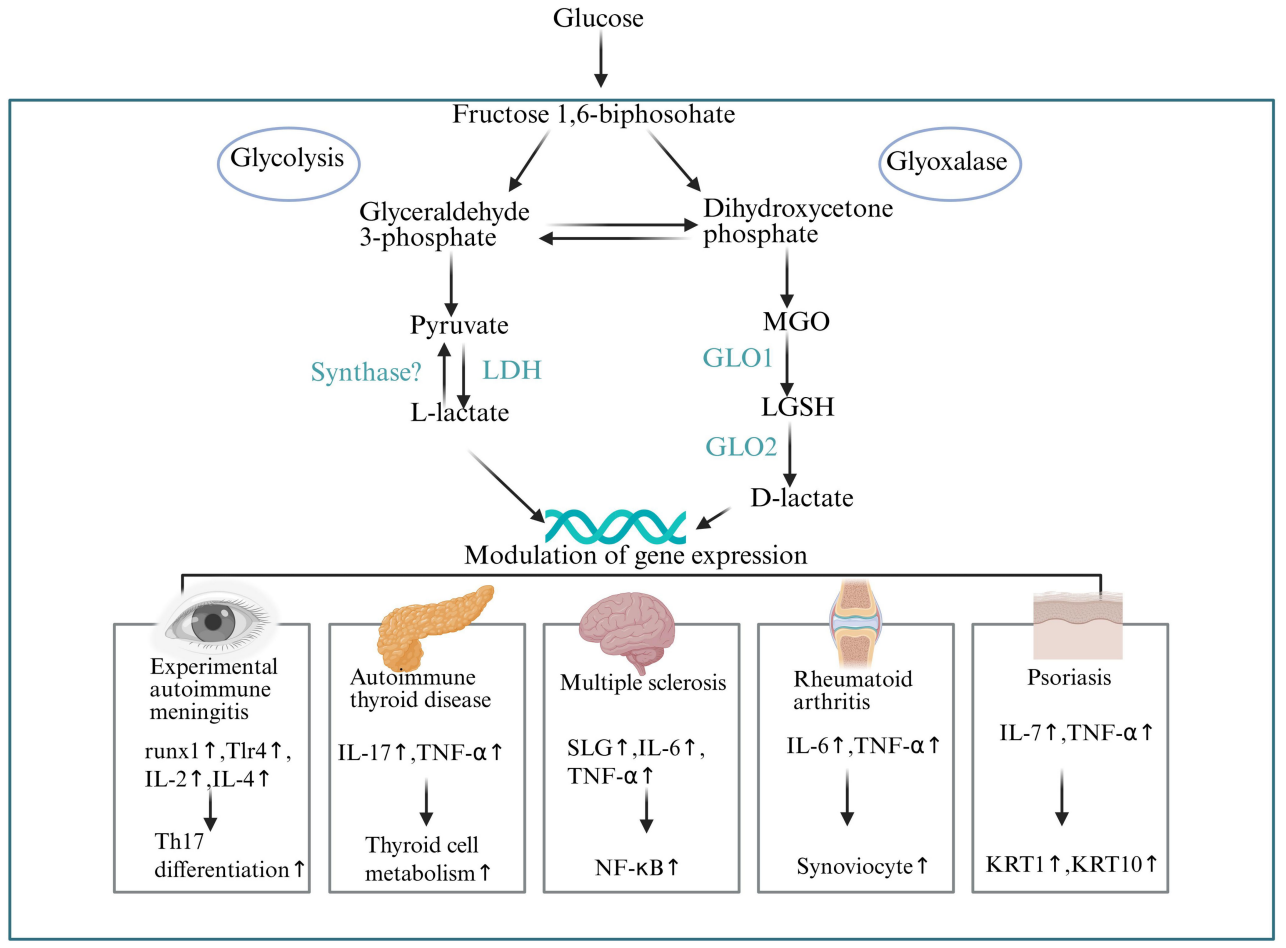
The role of lactylation in autoimmune diseases. This diagram reveals how glycolysis and the methylglyoxal pathway generate L-lactate and D-lactate, influencing autoimmune diseases. In experimental autoimmune encephalomyelitis, L-lactate boosts runx1 and Tlr4, spurring Th17 cell differentiation and inflammation. In autoimmune thyroid disease, lactate may alter thyroid cell metabolism, increasing IL-17 and TNF-α, damaging thyroid tissue. In multiple sclerosis, D-lactate adjusts SLG, IL-6, and TNF-α, activating NF-κB and worsening neuroinflammation. In rheumatoid arthritis, lactate promotes synoviocyte proliferation and releases IL-6 and TNF-α, intensifying joint issues. In psoriasis, lactate regulates IL-7 and TNF-α, causing abnormal keratinocyte proliferation and skin lesions. These insights highlight glucose metabolism’s role in autoimmune diseases, suggesting new treatment targets.

Lactate modification, as a crucial post-translational modification, significantly impacts the pathogenesis of numerous autoimmune diseases. Further elucidation of its mechanisms and exploration of potential therapeutic targets can offer new insights and methods for the clinical diagnosis and treatment of autoimmune diseases (**Figure 5**).

## 8 Potential and challenges of lactylation as a therapeutic target

In recent years, as the roles of lactylation across various diseases have been progressively elucidated, research on the regulatory factors governing lactylation has also achieved significant advances. These modulators primarily intervene in the “writers,” “erasers,” and lactate-metabolic pathways of lactylation, thereby tuning cellular functions and disease progression (**Table 2**).

**Table 2:** The role of lactate modification modulators.

Drug category	Drug name/target	Mechanism of action	Potential therapeutic effects	Research status/application prospects	References
“Writer” Inhibitors	C646(p300/CBP)	Inhibits p300/CBP acetyltransferase activity, indirectly suppressing histone lactylation.	Reduces lactate modification by inhibiting p300 activity	Demonstrated anti-tumor effects in multiple tumor models	(96)
	A-485(p300/CBP)	pecifically targets p300/CBP to inhibit lactylation	Delays tumor progression	Significant effects shown in breast cancer models	(45, 97)
	Curcumin	Reduces lactate modification by inhibiting p300 activity	Anti-inflammatory and anti-tumor properties	Lower selectivity but broad biological activity	(98)
Lactylation “Eraser” Activators	SIRT1 Activator (Resveratrol)	Activates SIRT1 to remove lactylation from α-MHC	Restores cardiac function	Significant effects in heart failure models	(99)
	HDAC Inhibitors (Trogustatin A, Vorinostat)	Modulates deacetylation/delactylation balance to indirectly affect lactate levels	Anti-tumor and anti-inflammatory	Demonstrated anti-tumor effects in multiple tumor models	(100)
Lactate Metabolic Pathway Modulators	Oxamate (LDH Inhibitor)	Inhibits LDH to reduce lactate production	Reduces lactate efflux by inhibiting MCT1	Demonstrated anti-tumor effects in multiple tumor models	(101)
	AZD3965 (MCT1 Inhibitor)	Inhibits MCT1 to reduce lactate efflux	Reduces lactate modification levels	Used in clinical trials for tumor therapy	(102)
Epigenetic Combined Modulators	p300/CBP Dual Inhibitors	Simultaneously inhibits lactylation and acetylation	Enhances anti-tumor effects	Significant effects shown in multiple tumor models	(103)
	Azacytidine (DNA Methylation/Demethylation Drug)	Combines with DNA methylation/demethylation drugs to enhance anti-tumor effects.	Reduces drug resistance associated with single-target therapies	Significant effects shown in multiple tumor models	(104)

### 8.1 Inhibitors of “writer” enzymes for lactylation

The catalytic enzymes responsible for lactylation have not yet been fully characterized. Nevertheless, accumulating evidence indicates that histone lactylation is mediated—at least in part—by classical histone acetyl-transferases such as p300/CBP or by newly identified lactyl-transferases. The p300/CBP inhibitor C646 suppresses histone lactylation, such as H3K18la, thereby attenuating pro-inflammatory gene expression in tumor cells (102). In pre-clinical models of melanoma and colorectal cancer, C646 treatment markedly suppressed tumor growth and heightened sensitivity to chemotherapeutic agents; however, its direct clinical application is hindered by side effects such as cardiotoxicity (103). A-485, a more selective small-molecule p300/CBP inhibitor, has demonstrated the ability to delay tumor progression by curbing lactylation and other histone modifications in breast-cancer xenograft models (12, 50). Natural products like curcumin (104) can likewise reduce lactylation by inhibiting p300 activity and have shown anti-inflammatory and anti-tumor efficacy in animal studies, yet their low selectivity and unfavorable pharmacokinetic profiles remain the principal obstacles to clinical translation.

### 8.2 Activators of lactylation “eraser” enzymes

Although enzymes that specifically remove lactylation have not yet been conclusively identified, histone deacetylases (HDACs) and certain sirtuins are thought to participate in de-lactylation. For example, SIRT1 has been shown to delactylate α-MHC in a heart-failure model (105),and its activator resveratrol improves cardiac function and reduces fibrosis in the corresponding mouse model. However, resveratrol has pleiotropic effects, so it remains unclear whether its therapeutic benefits are primarily attributable to delactylation. Broad-spectrum HDAC inhibitors such as trichostatin A and vorinostat (106) can indirectly alter lactylation levels by shifting the balance between deacetylation and delactylation. Notably, in the experimental autoimmune encephalomyelitis (EAE) model, vorinostat administered at 50 mg/kg/day reduced the clinical score from 4.2 to 2.1 and delayed disease onset by 4.5 days, but it also caused a global 3.2-fold hyperacetylation of histones H3 and H4, leading to immunosuppressive side effects—including a 38% decrease in CD4^+^ T-cell numbers and a 65% reduction in IL-2 production (12), These findings underscore the urgent need to develop tools that selectively target dedicated “delactylases”.

### 8.3 Regulators of the lactate-metabolic pathway

Targeting lactate production offers an upstream strategy for modulating lactylation. Inhibiting lactate dehydrogenase (LDH) or monocarboxylate transporters (MCT) decreases both lactate accumulation and lactylation levels. LDH inhibitors such as oxamate or FX-11 lower tumor-microenvironment lactate concentrations (107), In the collagen-induced arthritis (CIA) model, intraperitoneal treatment with the LDHA inhibitor sodium oxamate (100 mg kg^-1^ day^-1^) reduced intra-articular lactate by 60% and H3K18la by 50%; arthritis scores fell from 8.2 to 3.5, while TNF-α and IL-6 expression in joint tissue declined by 72% and 65%, respectively (108). MCT inhibitors, exemplified by AZD3965 (targeting MCT1), decrease lactate export and hence lactylation. In a phase I trial (NCT01791595), AZD3965 elevated blood lactate (> 8 mmol L^-1^) and caused metabolic acidosis in some patients (109),Systemic inhibition of lactate metabolism may, however, disturb normal energy homeostasis in muscle and brain, necessitating tissue-specific targeting strategies.

### 8.4 Epigenetic combination regulators

Dual-targeting strategies are currently under intense investigation. Molecules that simultaneously inhibit lactylation and acetylation—such as p300/CBP bifunctional inhibitors (110), or combinations with DNA methylation/demethylation drugs such as azacitidine are being explored to enhance anti-tumor efficacy (111). In pre-clinical myelodysplastic syndrome (MDS) models, co-administration of an HDAC inhibitor with the demethylating agent azacitidine produced synergistic effects, illustrating the promise of combinatorial PTM modulation. Such regimens, however, carry the risk of additive toxicities, demanding meticulous therapeutic design (108).

### 8.5 Emerging technologies for lactylation research

Comprehensive dissection of lactylation relies on the synergistic use of cutting-edge tools. Mass-spectrometry (MS)-based proteomics, coupled with anti-Kla antibody immunoprecipitation and high-resolution LC-MS/MS, currently underpins the discovery and mapping of lactylation sites (22). Quantitative strategies—label-free quantification (LFQ) or tandem mass tags (TMT)—accurately capture dynamic lactylation changes, for example during M1 versus M2 macrophage polarization (45). Beyond identification, functional validation is key: site-directed mutagenesis(K → R to mimic de-lactylation, K → Q to mimic constitutive modification) remains the gold standard. Computational biology, including machine-learning algorithms for lactylation-site prediction and bioinformatic tools linking histone lactylation to gene-expression signatures, is now indispensable for mining multi-omics datasets and generating testable hypotheses (112).

## 9 Crosstalk between lactylation and other PTMs in autoimmunity

In autoimmune settings, lactylation does not operate in isolation but participates in an intricate regulatory network with other PTMs, primarily through competition or cooperation at shared lysine residues. The most prominent crosstalk occurs between lactylation and acetylation, as both share the same “writers” such as p300/CBP and “erasers” (HDACs, sirtuins) (113). In SLE CD4^+^ T cells, H3K18la and H3K18ac dynamically balance each other on the same histone tail, jointly tuning key inflammatory pathways including the IFN response (13). This competitive interplay directly influences chromatin accessibility and transcription-factor recruitment. Beyond acetylation, lactylation functionally interacts with phosphorylation, methylation, succinylation, and more. In activated macrophages, lactylation at sites adjacent to STAT1 enhances its phosphorylation (p-STAT1), amplifying IFN-γ signaling and potentially exacerbating auto-inflammatory responses (114). On histones, H3K9 lactylation (H3K9la) and H3K9 trimethylation (H3K9me3) are usually antagonistic—activation versus silencing. This balance is disrupted in rheumatoid-arthritis synovial fibroblasts, sustaining a pro-inflammatory phenotype (115). Metabolite-driven crosstalk adds another layer: elevated succinate induces lysine succinylation (Ksucc) that competes with lactylation for identical or adjacent residues. In the EAE model, altered succinylation in CD4^+^T cells modulates lactylation-mediated Th17 differentiation, revealing metabolic cross-talk between distinct modifications (9). Understanding these complex PTM networks is crucial for developing therapies that precisely target lactylation without perturbing other essential modifications.

### 9.1 Summary and outlook

In recent years, lactylation has emerged as a novel post-translational modification with promising therapeutic potential for autoimmune diseases. Yet, translating this insight into clinical practice still faces formidable challenges. The foremost obstacle is that the underlying regulatory mechanisms remain incompletely understood: neither the dedicated “writer” nor the “eraser” enzymes have been definitively identified, which directly impedes the development of highly specific drugs. In addition, lactylation engages in extensive crosstalk with other post-translational modifications—such as acetylation, methylation, and crotonylation—further complicating selective therapeutic intervention (12), such as shared “writers” (p300) and “erasers” (HDACs and Sirtuins) (116). Consequently, current tools—p300 inhibitors, HDACi—are non-selective; inhibiting lactylation inevitably perturbs other vital PTMs, yielding unpredictable off-target effects and toxicity. Future efforts must therefore harness structural and chemical biology to create highly selective agonists/antagonists that discriminate lactylation from other modifications, especially acetylation. Examples include inhibitors that specifically target unique domains within p300 responsible for lactylation but not acetylation. Finally, existing modulators such as the natural product curcumin suffer from poor selectivity, pharmacokinetics, and bioavailability; the safety and efficacy of lactylation-targeting agents must be rigorously validated in advanced pre-clinical and clinical studies before they can reach the bedside.
